# The Impact of Routine Laundering on Ultraviolet Protection Factor (UPF) Values for Commercially Available Sun-Protective Clothing

**DOI:** 10.7759/cureus.42256

**Published:** 2023-07-21

**Authors:** Erika Fernau, Sammy M Ilyas, Erum N Ilyas

**Affiliations:** 1 Dermatology, Drexel University College of Medicine, Philadelphia, USA; 2 Research and Development, UVtec, LLC, King of Prussia, USA

**Keywords:** laundering, uv protection, uv protective clothing, upf, sun protective clothing

## Abstract

Background

Ultraviolet (UV) radiation has potentially harmful effects on the skin. Sunscreen products have historically focused on blocking UV-B radiation to prevent sunburns while efforts to block UV-A radiation have been lacking. UV protective clothing, rated by ultraviolet protection factor (UPF) values, has gained popularity as an alternative form of UV protection, offering a physical barrier against UV rays. However, concerns arise regarding the disclosure and sustainability of UV-protective textiles, as companies often do not disclose the methods used to achieve UV protection. The addition of chemical sunscreen additives to textiles raises questions about their potential release during laundering and their impact on sustained UV protection and environmental health. Further research is needed to understand the risks and benefits of these practices.

Methods

Seven garments from commercially available sun-protective brand names claiming UV protection were tested for UPF values. The garments were washed separately using cold water in commercially available detergent in cold water followed by drying on a low setting. UPF measurements were obtained at baseline and at intervals of 10 wash cycles until 50 wash cycles were completed.

Results

Two brands (Brands A and D) experienced a significant decrease in UPF value (70% to 78%) by the completion of 50 washes. Brand A disclosed the use of a nano-zinc additive in their garments while Brand D did not disclose the means of achieving UV protection. In comparison, five brands (Brands B, C, E, F, G) maintained relatively stable UPF values throughout the 50 washes. The comparison between Brand A and Brand G, who disclosed their UV protection methods, showed that Brand A gradually decreased in UPF value throughout the washes while Brand G remained stable.

Conclusion

The findings suggest that textile compositions with UV finishes may lose their UPF effectiveness during laundering by loss of the finish used over time or the textile integrity could be affected. This raises questions about the necessity of adding these UV finishes if there are fabrics that can maintain their UPF values without them.

## Introduction

The potentially damaging effects of ultraviolet (UV) radiation on the skin have been well-documented. Premature aging of the skin, photosensitive conditions, and pigmentary disorders, as well as a well-studied link to skin cancers, such as squamous cell carcinoma, basal cell carcinoma, and melanoma, have been established [1]. Over the past two decades, sunscreen products have been increasingly recommended for this purpose. Sun protection factor (SPF) values are determined by the ability of skincare products to block UV-B radiation linked to sunburns, which have been linked to skin cancer development [2]. However, the ability of a sunscreen product to block UV-A radiation has been historically lacking, at least partially because these products were originally designed to prevent sunburns by allowing UV-A radiation through to permit pigmentation of the skin based on commercial interest in this cosmetic effect [3].

Increasingly, consumers are seeking UV-A protection given its link to autoimmune, photosensitive disorders, medication reactions, and premature aging of the skin. Sunscreen products have added the term “broad spectrum” to indicate the presence of ingredients that can block UV-A; however, this term is not a regulated claim with the claim offering widely variable percentages of UV-A blockage [4]. The use of sunscreen products of varying types carries nuances with regards to the amount applied, spent time on application, reapplication, and sustained UV protective activity of the product [5]. Therefore, the impractical aspects regarding routine sunscreen use necessitate other forms of UV protection. 

UV protective clothing is increasing in popularity in recent years with more consumers seeking options [6]. Although intuitively, most fabrics will offer some degree of UV protection by creating a physical barrier between the skin and UV sources, such as the sun, UV protective clothing is rated based on testing for the amount of UV-A and UV-B blockage provided by the textile. When tested, an ultraviolet protective factor (UPF) rating is assigned to the clothing to indicate if the textile has good, very good, or excellent UV protection if the textile blocks a minimum of 93% of UV rays. UPF materials can achieve UV protection via several natural properties of the fibers used to construct the textile, in addition to aspects of textile construction, coloration, and composition. An additional means to artificially achieve added UV protection to textiles is to add sunscreen additives to the construction process either by embedding metal oxides into the fibers during the construction process or coating the textiles with chemical sunscreen additives [7]. UV textiles can protect the skin by reflecting, absorbing, and scattering solar wavelengths.

The American Society for Testing Materials (ASTM), has assigned standard practice to label garments with UPF values. The ASTM 6544 is a standard protocol that designates the UPF value assigned to a garment as the lowest UPF value obtained after the garment is subjected to 40 launderings to simulate “consumer use for two years” [8]. During UPF testing, UV-A and UV-B rays are passed five times in different directions through the fabric to simulate sunlight through different aspects of the garment with an average obtained. Meanwhile, SPF measures only the transmittance of UV-B rays and is based on the time the sunscreen is applied [9]. 

Several companies market UPF clothing. These companies do not consistently disclose how the textiles used in their garments achieve UV protection. For those made with UV finishes embedded in the fibers or coating on the textiles raises the question of the potential for these to wash out in routine laundering and potentially impact the sustained UV protection offered by these textiles or potentially impacted by sweating or swimming. Although there are studies on the absorption of chemical sunscreen ingredients when applying sunscreen products [10], we could not find any studies on the absorption of chemical sunscreen additives in clothing into our bloodstream. UV protection achieved through the fiber composition and construction of the textile has been found to increase after washing likely as a result of fabric shrinkage [11]. The addition of UV chemical finishes to textiles is likely to increase the inherent UPF of textiles used. The risks and benefits of this practice are unclear given the unclear impact of these finishes if released from the garment during laundering, sweating, or swimming as we did not come across any studies evaluating the finish once used. 

As it is unclear if UV finish chemicals have the potential to release from textile materials, penetrate the skin, and enter the bloodstream, there is the potential to impact human health and the environment [12]. With potentially 95% of our body surface area in contact with textiles during the course of a day or night, there is a concern about exposure to textile additives. Although data on UV finishes in textiles with an impact on the skin are lacking, some textile additives for other textile properties have been shown to interact with the skin. For example, formaldehyde in clothing has been linked to skin irritation [13]. With research suggesting that certain dyes and finishes can impact agricultural productivity, and lower oxygen content in the waterways, leading to aquatic life and plant death, this leads to concerns for the environment as well [14]. Moreover, there has been a growing focus on the safety of SPF products, leading to recommendations to refrain from using nanoparticles in sunscreens due to their proven harmful effects on coral reefs and marine life [15,16].

We sought to review a range of clothing items offered by common brand names that claim to offer UV protection via their textiles to determine the change in UPF from purchase and through 50 laundering cycles to determine if a change in UPF was noted. If a decrease in UPF is noted, our hypothesis is that UV sunscreen additives in textiles may decrease over routine use and potentially be released to the environment or that the textiles may lose their integrity compromising their ability to offer UV protection over time.

## Materials and methods

Seven common brand names for sun-protective clothing were chosen based on marketing claims made about the UPF of garments. The branding labels were removed from the garments to blind the test administrator, with each garment assigned a letter value from A through G. The compositions and finishes of these garments as determined by garment labeling and review of brand websites are displayed in Table 1.

**Table 1:** UPF values, composition, and disclosed UV treatments of garments tested UPF: ultraviolet protection factor; UV: ultraviolet

Brand Label	UPF claim	Composition	UV chemical finish
A	50+	Cotton/Viscose from Bamboo/Elastane blend, % not disclosed	Nano zinc
B	20+	68% Viscose from Bamboo / 29% Polyester / 3% Spandex	No UV treatments used
C	50	55% Polyester, 39% Nylon, 6% Spandex Jersey	Not specified
D	40 to 50+	92% Polyester, 8% Spandex	Not specified
E	50+	100% Polyester	Not specified
F	50+	90% Polyester/10% Spandex	Not specified
G	50+	89% Polyester, 11% Spandex	No UV treatments used

These garments were tested for initial UPF values on purchase of the garment with the UV 2000F Ultraviolet Transmittance Analyzer (LabSphere, North Sutton, NH). The UV 2000F Ultraviolet Transmittance Analyzer measures diffuse transmittance in the ultraviolet wavelength region from 250-450 nm to determine UPF values. After passing the garment through the analyzer in five different directions, the device determines a mean UPF value to assign to the garment along with providing UV-A and UV-B transmittance values. 

After obtaining the initial UPF value, each of the seven garments was washed separately in the cold water setting using commercially available detergent (Tide Detergent, Procter & Gamble, Cincinnati, Ohio). They were then dried in the normal setting without the use of fabric softener to reduce the impact that fabric softener could potentially have on UPF values. Separate washing was performed to avoid the transfer of any possible chemical finishes on the garments between garments to reduce the impact on testing. UPF measurements were obtained after 10 washes, 20 washes, 30 washes, 40 washes, and 50 washes. The results are shown in Table 2.

**Table 2:** UPF values obtained for each garment after 10 laundering cycle intervals UPF: ultraviolet protection factor

Name	Zero Washes	10 Washes	20 Washes	30 Washes	40 washes	50 Washes
Brand A	567	222	312	225	174	126
Brand B	25	23	27	28	26	20
Brand C	84	99	88	94	104	98
Brand D	492	538	160	199	97	151
Brand E	78	187	106	112	127	78
Brand F	51	90	75	75	57	83
Brand G	2000	2000	2000	2000	2000	2000

## Results

**Figure 1:**
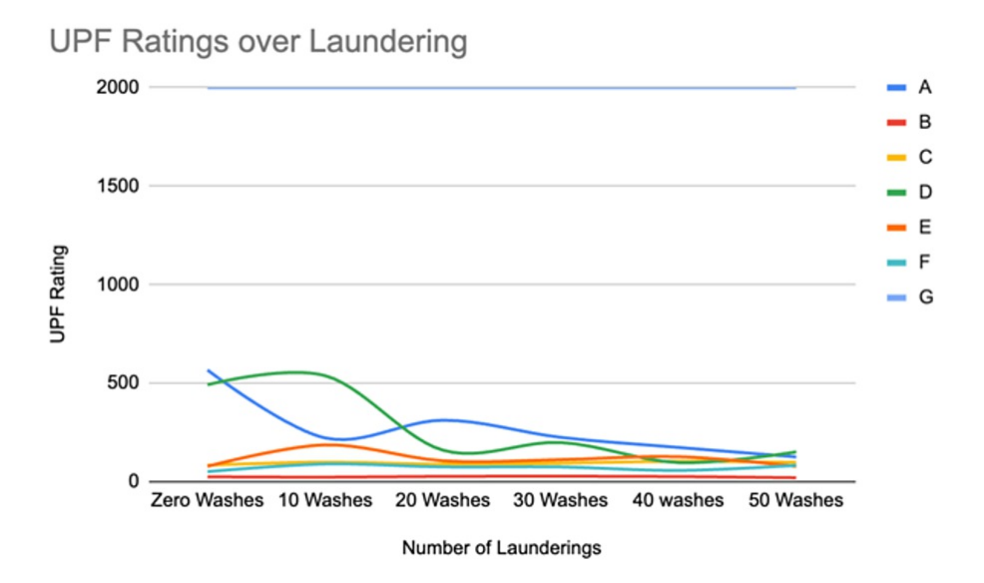
Change in UPF ratings for Brands A through G through 50 laundering cycles UPF: ultraviolet protection factor

**Figure 2:**
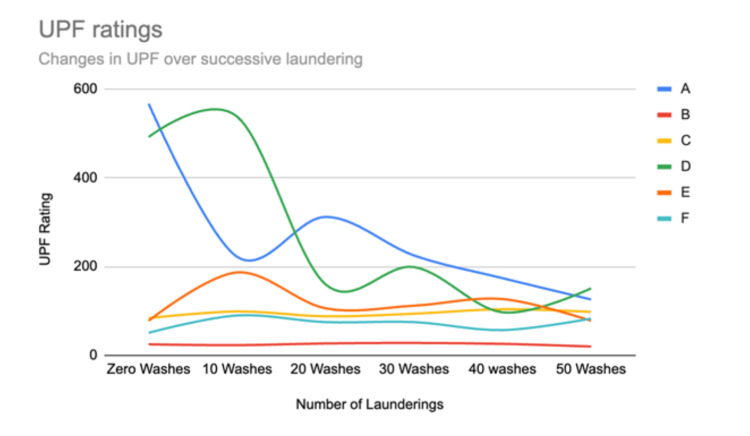
Change in UPF ratings for Brands A through F through 50 laundering cycles

Descriptive statistics were used to review the data (Figures 1, 2). The data shows that two brands (Brand A and Brand D) decreased their UPF value by 70% to 78% from their initial values through the completion of 50 laundering cycles. Brand A disclosed the use of a nano-zinc additive to the textiles utilized in their garments while Brand D had no disclosure of the means of achieving UV protection in the textiles used. Five brands (Brands B, C, E, F, G) held relatively stable UPF values through 50 washes with fluctuations in values while ultimately either increasing the UPF or staying stable relative to the initial UPF value obtained. Of these brands, Brand B and Brand G disclosed that no UV chemical finishes were utilized in the textiles chosen for these garments.

Without disclosure from Brand C, Brand D, Brand E, and Brand F as to how UV protection was achieved, we can only compare Brand A, Brand B, and Brand G with disclosures for UPF changes based on laundering with and without UV finishes. Brand A discloses a nano-zinc finish while Brand B and Brand G state no UV chemical finish was used. Brand A was able to stay above a UPF of 50 for 50 laundering cycles; however, the UPF did gradually decrease through the washes ultimately dropping to 78% of its original UPF value by the end of 50 laundering cycles. Brand B with no UV finishes used fluctuated within 20% of its original UPF value through 50 laundering cycles. Brand G with no UV finishes used stayed at the maximal UPF value assignable of 2000 through 50 laundering cycles.

## Discussion

We found several companies that market UPF clothing where product labeling revealed compositions of textiles for fibers chosen in the textile without revealing the method by which these textiles achieved UV protection. Product labeling for sunscreen products applied to the skin has an ingredient label that permits us to find this data easily; however, clothing is only labeled for fiber composition and not the presence of chemical finishes used during the manufacturing process. Further website reviews did not consistently disclose how the textiles used in their garments achieved UV protection.

Brand A revealed the use of a UV finish consisting of nano-zinc. Brand A had a reduction of the UPF factor throughout the laundering process by 78%. In spite of this reduction, the UPF value did stay above 50, consistent with the UPF labeling claim. 

Brand B was marketed as UV protective, with UPF values never exceeding 30. Brand B stated that no UV finishes were used and marketed the use of bamboo in the textile as naturally sun-protective. The UPF value stayed relatively stable through 50 washes although it never exceeded 30.

Brands C, E, and F maintained relatively stable UPF values that stayed in excess of 50 throughout the 50 laundering cycles. These brands did not reveal the use of UV finishes.

Brand D maintained UPF values well above 50 throughout 50 laundering cycles. Brand D did not reveal the presence of UV finishes; however, the testing revealed a similar pattern for reducing UPF values through 50 washes.

Brand G with a similar fiber composition to Brand F had substantially higher UPF values at the highest value attainable of 2000 indicating 99.5% UV-A and UV-B transmittance blockage. This brand sustained this UPF of 2000 through 50 laundering cycles and stated that no UV finishes were used in the manufacturing process of this textile.

Brand A as the only brand revealing the use of a UV finish in the textile used had a reduction of the UPF factor throughout the laundering process by 78%. Opposingly, Brand B and Brand G without any UV finish maintained the overall UPF integrity through 50 laundering cycles. This implies that either some of the UV finish is lost throughout the laundering process, reducing the overall UPF factor of these compositions or that the integrity of the textile was compromised by the laundering process for Brand A. Opposingly, the compositions without any UV finish maintained the overall UPF integrity through 50 laundering cycles. This implies that either some of the UV finish is lost throughout the laundering process reducing the overall UPF factor of these compositions or that the integrity of the textile was compromised by the laundering process for Brand A.

Brand B without chemical finishes used maintained the UPF within 20% of its original value through 50 laundering cycles. Brand G maintained maximal UPF ratings of 2000 through 50 laundering cycles. This implies that the UPF values of these textiles were dictated by the properties and integrity of the textile itself, such as composition and construction, given that no additional UV finish was used in these textiles.

Therefore, the question arises as to how a UV chemical finish offers a benefit to textiles for added UV protection and whether the risk of depletion of this finish can compromise the benefits offered over time. If the UPF diminishes, the chemicals added to the textile to confer added UV protection may be released to the water supply through the laundering process or expose our skin to these products via sweating or swimming potentially releasing these agents. In some cases, these finishes may make garments more wearable for the user. However, if there are fabrics that can maintain their UPF integrity throughout laundering without any finishes, it could be argued that these finishes may not be necessary in the first place.

The data also reveal that the fiber composition of textiles is not enough data to determine the potential for UV protection from a garment, given the similar compositions between Brand F and Brand G with substantial variation in UPF values. Brand F sustained UPF values 51 to 90 through 50 laundering cycles while Brand F stayed at the maximal UPF assignable of 2000 through 50 laundering cycles.

Of note, skincare products carry ingredient labels allowing the user to review prior to use, whereas only 42.8% of brands tested revealed to the consumer how UPF was achieved.

There were several limitations of this study. Although many well-known brands may include UPF clothing, there are not many sun-protective clothing brands that market themselves exclusively based on the UPF value of their clothing. "Sun-protective clothing" is not a clearly defined term to indicate the amount of UV protection offered by the clothing as was evidenced by Brand B. Without clearly defined guidelines for using this terminology and varying sources for this type of clothing, we were limited to seven brands that clearly stated the value proposition of their brands as sun protection. Without access to the chemical finishes used in the manufacturing process of the four brands evaluated, it was difficult to assess the impact of laundering on the presence or absence of a UV finish.

## Conclusions

The question we posed was whether a UV finish added to fabric could actually be released over time through the laundering process. This could potentially lead to added exposure of these agents to our skin as well as to the environment through the laundering process if these chemicals enter the waterways. The results of this study reveal that the UPF of textiles appears to remain overall stable through laundering, with the exception of two textiles tested, one of which disclosed the use of nano-zinc in their textiles and the other without disclosure on how UPF was attained. With only knowledge of the use of nano-zinc in one textile with a reduction in UPF noted, the question as to whether this finish is depleted through the laundering process arises. Further studies are needed to determine the presence of these UV-protective agents in wastewater post laundering as well as disclosures from sun-protective clothing brands as to the source of UV protection in textiles used.
